# Probing the Potential Mechanism of Quercetin and Kaempferol against Heat Stress-Induced Sertoli Cell Injury: Through Integrating Network Pharmacology and Experimental Validation

**DOI:** 10.3390/ijms231911163

**Published:** 2022-09-22

**Authors:** Dian-Long Liu, Si-Jia Liu, Su-Qin Hu, Yu-Cai Chen, Jian Guo

**Affiliations:** Department of Physiology, College of Traditional Chinese Medicine, Beijing University of Traditional Chinese Medicine, No. 11, East Beisanhuan Road, Chaoyang District, Beijing 100029, China

**Keywords:** flavonoids, testis, heat stress, anti-oxidation, anti-inflammation, blood testis barrier

## Abstract

Quercetin and kaempferol are flavonoids widely present in fruits, vegetables, and medicinal plants. They have attracted much attention due to their antioxidant, anti-inflammatory, anticancer, antibacterial, and neuroprotective properties. As the guarantee cells in direct contact with germ cells, Sertoli cells exert the role of support, nutrition, and protection in spermatogenesis. In the current study, network pharmacology was used to explore the targets and signaling pathways of quercetin and kaempferol in treating spermatogenic disorders. In vitro experiments were integrated to verify the results of quercetin and kaempferol against heat stress-induced Sertoli cell injury. The online platform was used to analyze the GO biological pathway and KEGG pathway. The results of the network pharmacology showed that quercetin and kaempferol intervention in spermatogenesis disorders were mostly targeting the oxidative response to oxidative stress, the ROS metabolic process and the NFκB pathway. The results of the cell experiment showed that Quercetin and kaempferol can prevent the decline of cell viability induced by heat stress, reduce the expression levels of HSP70 and ROS in Sertoli cells, reduce p-NF-κB-p65 and p-IκB levels, up-regulate the expression of occludin, vimentin and F-actin in Sertoli cells, and protect cell structure. Our research is the first to demonstrate that quercetin and kaempferol may exert effects in resisting the injury of cell viability and structure under heat stress.

## 1. Introduction

Infertility is an important cause of marital emotional problems, family anxiety, family depression, and social psychological disorders. Infertility is a recognized problem in the field of world health. Male factors account for a high proportion, accounting for approximately 40–70% of infertility factors. Male infertility is a multifactorial disease, and many complications contribute to its diagnosis. Heat stress is a major problem that men are increasingly facing in the process of global warming and modernization [1]. The high-temperature environment around the testis is closely related to the decline of sperm quality [2,3]. The incidence rate of heat stress-induced spermatogenesis disorder and related mechanisms have been reported by many previous studies [4,5]. Exploring the mechanism of heat stress-induced spermatogenesis disorder is helpful to develop new and effective treatments to reduce the incidence rate and severity of male infertility caused by increasingly serious heat stress environments.

Oxidative stress is one of the most concerned pathological mechanisms of heat stress-induced spermatogenesis disorder [6,7]. The most important mechanism is the excessive production of reactive oxygen species (ROS). Overproduction of ROS (arising either from a mitochondrial electron-transport chain or excessive stimulation of NAD(P)H) results in oxidative stress, a deleterious process that is an important mediator of damage to cell structures, including lipids, membranes, proteins, and DNA. The accumulation of oxidative stress products due to high temperature leads to an increase in the expression of heat shock proteins (HSPs) [8]. HSPs are a group of highly conserved proteins produced by the body after external or internal physical and chemical stimulation [9]. According to molecular weights, HSPs can be divided into seven families as follows: HSP110, HSP90, HSP70, HSP60, Hsp40, small HSP (sHSP), and ubiquitin. Hsp70 is the most widely and thoroughly studied HSP. HSP70 accelerates the elimination of ROS by cells by reducing ROS production and increasing superoxide dismutase (SOD) levels [10,11]. SOD is a general enzyme present in organisms requiring oxygen, and it catalyzes the conversion of superoxide into oxygen and hydrogen peroxide. Through their activity, SOD enzymes control the levels of ROS, thus limiting the potential oxidative stress toxicity of these molecules and controlling the cellular dysfunction caused by their abnormal signal transduction.

The NF-κB signaling pathway plays an important role in the inflammatory response, oxidative stress, cell proliferation, and apoptosis. Studies have reported that NF-κB regulates TNF-α, AR, FasL, and other related genes of the inflammatory response and apoptosis function in testis [12]. The NF-κB signaling pathway is easily activated by ROS, and its activation degree is regulated by antioxidants, such as SOD. ROS activates IKK, which phosphorylates IκB, releasing the NF-κB p65/p50 dimer. The NF-κB p65/p50 dimer translocates into the nucleus for transcription and expression, activating the NF-κB signaling pathway [13], which induces the inflammatory response and leads to spermatogenesis disorder [14,15].

Sertoli cells (SCs) are the only somatic cells in the seminiferous tubules that contact germ cells, and they play a vital role in the nutrition, support, and protection of sperm during its development, maturation, and transport to the epididymis [16,17]. Injury to the structure and function of SCs and the blood-testis barrier (BTB), which is mainly composed of SCs, affects the normal spermatogenesis process to a great extent, leading to serious spermatogenesis disorders [2,18]. Studies have shown that when the testis is given local heat shock treatment, the gap between SCs and sperm cells increases, the structure of seminiferous tubules becomes loose, and the expression of occludin is downregulated [19,20]. Occludin is involved in the intercellular connection, signal transduction, and transcriptional regulation in the BTB. Occludin is an important structural protein that maintains the normal function of the barrier [21]. Vimentin is the main component of the intermediate filament of the supporting cytoskeleton, and it mainly plays an “anchoring role” in the BTB [22]. F-actin is the main component of cytoskeleton microfilaments, which not only maintain the morphology of supporting cells but also form a network structure with tight junction proteins, generating a tight junction contraction system [23,24]. When the expression and structural localization of occludin (structural protein), vimentin (skeletal protein), and F-actin are abnormal, the tight junction complex is destroyed, and the BTB is damaged, resulting in spermatogenic cell apoptosis [25].

Although great progress has been made in the investigation and treatment of spermatogenic disorders in the field of modern medicine, the primary treatment route adopted by most men still focuses on traditional therapies based on medicinal plants, that is, the active ingredients of traditional herbs. The efficacy of these plant-active substances mainly depends on their preparation methods, including decoctions, extracts, and semipurified compounds [26]. At present, common dietary supplements include vitamin C, melatonin, and other substances [27,28]. Many studies have reported that flavonoids, such as quercetin and kaempferol, have good antioxidant and anti-inflammatory properties [29]. Quercetin and kaempferol are contained in common fruits and vegetables, and they are enriched in traditional Chinese medicines, such as medlar, dodder, and raspberry, which inhibit oxidative stress and have anti-inflammatory effects [30,31]. However, the effects and mechanisms of quercetin and kaempferol on heat stress-induced SC injury have not been reported.

With the rapid development of bioinformatics, network pharmacology has become a powerful tool to explore the functions of complex compounds [32]. Network pharmacology can effectively explore multi-component, multi-target, and multi-channel complex compounds. The multi-target components of compounds may be the key material basis of their effects. However, the relationship of the main components of quercetin and kaempferol with their pharmacological effects is not clear. The present study aimed to elucidate the molecular mechanism of quercetin and kaempferol by analyzing the bioactive components of quercetin and kaempferol followed by the application of network pharmacology to further study the correlation between potential protein targets and central signal pathways related to male spermatogenic disorders. In addition, in vitro experiments further verified the potential molecular mechanism of quercetin and kaempferol against heat stress-induced SC injury predicted by network pharmacology methods. The present findings will greatly promote further research on medicinal plants that improve male reproductive health.

## 2. Results

### 2.1. Target Identification

**Figure 1:**
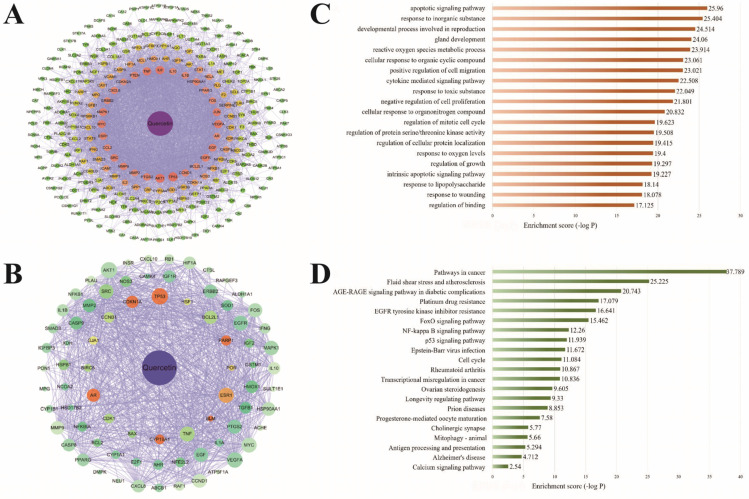
The main active components of quercetin and the prediction of biological targets for the treatment of spermatogenesis disorders (PPI network diagram of quercetin target (**A**), PPI network diagram of quercetin intervention spermatogenesis disorder target (**B**), go enrichment analysis of quercetin intervention spermatogenesis disorder target (**C**), and KEGG pathway enrichment analysis of quercetin intervention spermatogenesis disorder target (**D**)).

A total of 1191 potential targets of spermatogenesis disorders were collected through the online databases, and 78 targets of quercetin intervention in spermatogenesis disorders were obtained through Venn diagram analysis. The protein-protein interaction (PPI) network diagram of quercetin intervention in spermatogenesis disorders was constructed. Greater degree values of the target in the network indicated greater node radiuses, greater score values, and warmer colors (Figure 1). A total of 262 effective monomer targets of quercetin were collected (Specific information on gene targets can be found in Table S1 in the Supplementary Material). According to the analysis of the Metascape platform, the targets of quercetin intervention in spermatogenesis disorders were mostly enriched in oxidative response to oxidative stress, ROS metabolic process, regulation of the apoptotic signaling pathway, cellular response to external stimulus, regulation of cellular protein localization, and other biological processes. The KEGG pathway enrichment analysis of quercetin intervention in spermatogenesis disorders mainly focused on the FOXO signaling pathway and NF-κB signaling pathway.

A total of 159 effective monomer targets of kaempferol were collected (Specific information on gene targets can be found in Table S2 in the Supplementary Material). Analysis of the Venn diagram obtained 39 targets of kaempferol in the intervention of spermatogenesis disorders. According to the analysis of the Metascape platform, the targets of kaempferol intervention in spermatogenesis disorders were mostly enriched in the cellular response to oxidative stress, ROS metabolic process, regulation of the apoptotic signaling pathway, steroid metabolic process, cellular response to external stimuli, and other biological processes. The KEGG pathway enrichment analysis of kaempferol intervention in spermatogenic disorders mainly focused on mitophagy-animal, the HIF-1 signaling pathway, the NF-κB signaling pathway, and other oxidative stress or inflammatory response pathways.

### 2.2. Effects of Quercetin and Kaempferol on SC Viability and Heat Shock Protein Expression after Heat Stress

The effect of the dose gradient of quercetin and kaempferol on the viability of SCs indicated that both quercetin and kaempferol treatment significantly improved cell viability after heat stress. The effects of quercetin and kaempferol on cell proliferation activity showed corresponding changes with different concentrations. Compared to the control group, HSP70 expression was significantly increased after heat treatment in SCs in the model group (*p* < 0.001), and quercetin and kaempferol significantly reduced the expression of HSP70. HSP70 is one of the standard proteins expressed by SCs after heat stress, and its expression changes rapidly. HSP70, as a molecular chaperone, improves cell stress response, antioxidation effects, and antiapoptotic effects [33]. Previous studies have shown that after heat stress, the body enhances its tolerance to heat stimulation by increasing the expression of HSP70 [15,34]. However, HSP70 has the limited protective ability for the body. Excessive HSP70 destroys cell membranes and causes cell injury [35,36]. The decrease in HSP70 expression in cells treated with quercetin and kaempferol indicated that these compounds aid in resisting heat stress and blocking cell injury caused by the excessive increase of HSP70 to a certain extent.

### 2.3. Effects of Quercetin and Kaempferol on the Expression of ROS and SOD in SCs after Heat Stress

The fluorescence intensity of DCFH-DA in cells is directly proportional to the level of ROS content with ROS indicated by green, fluorescent particles in the cytoplasm. Darker particle colors and denser particle distribution indicate higher ROS contents. In the control group, the distribution of ROS was sparse, and the ROS content was low. Compared to the control group, the content and intensity of ROS were significantly higher in the model group. The content of ROS in the quercetin and kaempferol treatment groups was significantly lower than that in the model group, and the intensity of ROS in these treatment groups was similar to that in the control group.

After heat treatment, the SOD activity of the model group decreased compared to that of the control group, and the average SOD activity of the quercetin and kaempferol groups increased compared to that of the model group. Thus, these findings indicated that quercetin and kaempferol treatments reduce the heat stress-induced increase in ROS in SCs and increase the SOD content, which helps resist ROS to reduce the damage of oxidative stress to cells.

### 2.4. Effects of Quercetin and Kaempferol on the Expression of p-IκB and p-NFκB-p65 in SCs after Heat Stress

After heat treatment, the p-IκB level in the model group was significantly increased compared to the model group. Compared to the model group, the level of p-IκB in the quercetin and kaempferol treatment groups significantly decreased. NF-κB-p65 translocation from the cytoplasm to the nucleus is a marker of NF-κB activation. NF-κB p65 in the control group was evenly distributed in SCs, while NF-κB p65 in the model group was concentrated in the nucleus. Compared to the model group, the nuclear translocation of NF-κB p65 in the quercetin and kaempferol groups was significantly decreased. The phosphorylation level of NFκB-p65 in the model group was significantly higher than that in the control group. Compared to the model group, the quercetin (1 μM) and kaempferol (1 μM) treatment groups had significantly decreased p-NFκB levels. Thus, these data indicated that treatment with quercetin and kaempferol reduces the expression of p-NFκB and p-IκB in SCs induced by heat stress as well as reduces nuclear translocation of NF-κB-p65, thereby suppressing cellular inflammatory pathways.

### 2.5. Effects of Quercetin and Kaempferol on Occludin, Vimentin, and F-actin in SCs after Heat Stress

Compared to the control group, occludin expression in SCs in the model group was diminished, while quercetin and kaempferol protected occludin against heat stress in SCs. After heat treatment, occludin expression in the model group was significantly decreased compared to the control group, and occludin expression in the quercetin and kaempferol treatment groups was significantly increased compared to the model group, which was consistent with the immunofluorescence localization of occludin.

Because downregulation of occludin expression is often accompanied by abnormal tight junction function, occludin is often regarded as an indicator of the function and permeability of the BTB. Under normal physiological conditions, SCs regulate the expression and localization of occludin in SCs by secreting cytokines. Under the stimulation of oxidative stress and inflammatory response, the activation of signaling pathways, such as the NF-κB pathway, results in the secretion of too many cytokines, which downregulates occludin [37], resulting in the internalization of closing proteins on the cell surface, the formation of cell gaps, and the improvement of BTB permeability, ultimately reducing the expression of inhibin (INH) secreted by supporting cells and destroying the function of supporting cells [21,38,39].

The cytoskeleton is composed of microtubules, microfilaments, and intermediate filaments, which connect with tight junction proteins, thereby participating in the formation of desmosomes and hemidesmosomes as well as playing a role in maintaining cell shape, material exchange, positioning organelles, and assisting sperm release in SCs [40,41]. Destruction of the cytoskeleton often leads to disorder of spermatogenic epithelium and detachment of spermatogenic cells [38,42].

Vimentin is the main component of intermediate filaments, which form a structural network around the nucleus, maintain the morphology of cells, maintain the morphology of nuclei, connect with the nuclear membrane in many places, and support the adhesion, signal exchange, material transport, and other functions between cells and spermatogenic cells. After heat stress treatment, vimentin expression was decreased and showed abnormal distribution in SCs, which may destroy some functions of the BTB and hinder the spermatogenesis process. Treatment with quercetin and kaempferol resisted these negative effects.

Microfilaments are polymers of actin, mainly composed of F-actin, which are polymerized by two parallel G-actin filaments, and the microfilaments maintain the dynamic stability of the cell structure through the balance of polymerization and dissociation [31]. After heat stress, the F-actin around the nucleus was blurred and concentrated at the structural edge in the model group. Compared to the model group, the changes in F-actin structural edge concentration in the quercetin and kaempferol groups were improved. After treatment with 0.1 μM quercetin, the F-actin structural edge concentration was similar to that of the control group, which indicated that quercetin resisted damage to the F-actin structure and function of SCs induced by heat stress.

## 3. Discussion

**Figure 2:**
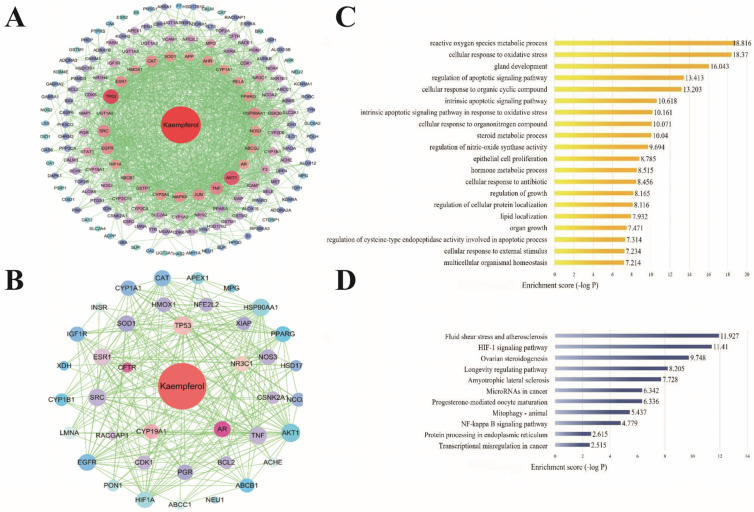
Main active components of kaempferol and prediction of biological targets for the treatment of spermatogenic disorders (PPI network diagram of kaempferol target (**A**), PPI network diagram of kaempferol intervention disease target (**B**), go enrichment analysis of kaempferol intervention disease target (**C**), and KEGG pathway enrichment analysis of kaempferol intervention disease target (**D**)).

In recent years, many studies have reported on the effect of flavonoids in botanical drugs on male reproductive system diseases. Flavonoids have significant effects on regulating reproductive endocrine, testicular, and epididymal development as well as testicular oxidative stress and inflammatory response [43]. To predict the potential mechanism of quercetin and kaempferol in the treatment of spermatogenic disorders, we performed GO enrichment analysis on 78 potential targets of quercetin and 39 potential targets of kaempferol. As shown in Figure 1C and Figure 2C, the 10 most significantly enriched items were mainly related to the metabolic process of ROS, cell response to oxidative stress, apoptosis, and gland development. Relevant studies have shown that the occurrence and development of spermatogenic disorders are related to cell dysfunction and damage as well as oxidative stress, indicating the importance of the related pathways of cellular oxidative stress response to quercetin and kaempferol. In addition, KEGG target enrichment analysis [44] showed that among the ten potential pathways of quercetin and kaempferol in the treatment of spermatogenic disorders, in addition to cancer and vascular atherosclerotic diseases, the NF-κB inflammatory signaling pathway was the most relevant, which was also highly related to cytokine-mediated signaling pathway in the GO enrichment analysis (Figure 1D and Figure 2D). However, it should be noted that investigating the effect of quercetin and kaempferol on spermatogenesis disorders only at the level of network pharmacology is limiting. At present, network information technology is not comprehensive, and the source of the database itself has a certain effect. For example, databases contain a large number of target predictions related to cancer research hotspots, and issues, such as reproductive development and gland development, were not included in the objectives of this study. Therefore, the present study synthesized these common targets with rich biological functions. However, considering that the targets are closely related to supporting cells, subsequent experiments are required to determine the oxidative stress and inflammatory signaling pathways involved in the most critical pathological mechanism of heat stress-induced spermatogenesis disorders. Interestingly, according to the results of another network pharmacology study, the antioxidant and anti-inflammatory effects of quercetin are mediated by the interference of signal transduction pathways (including AP-1 and NF-κB) [45]. In addition, quercetin improves the testicular structure damage induced by environmental toxins [46]. Other studies have shown that quercetin strengthens the intestinal epithelial barrier by inducing a significant increase in tight junction proteins [47]. Therefore, we believe that it is appropriate and reliable to design experimental validation of heat stress-induced SC injury by integrating network pharmacological target prediction and literature research to explain the therapeutic effect of quercetin and kaempferol.

**Figure 3:**
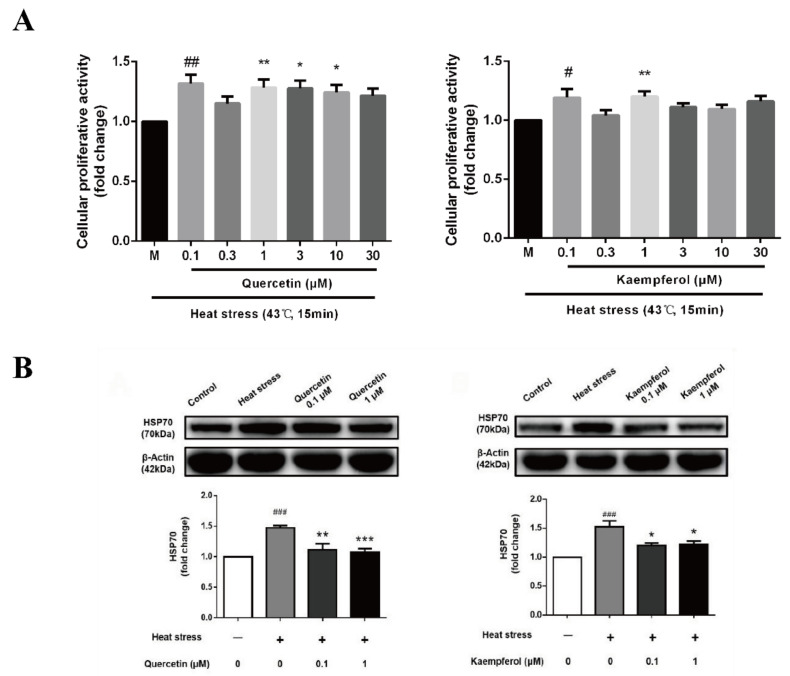
**Effects of quercetin and kaempferol on the activity and HSP70 expression of Sertoli cells induced by heat stress** (Analysis of cellular proliferative activity of Sertoli cell (**A**), HSP70 expression in Sertoli cell by Western blotting (**B**). Data are shown as mean ±SEM (*n* = 6 per group). (# *p* < 0.05, ## *p* < 0.01, ### *p* < 0.001, compared with the control group; * *p* < 0.05, ** *p* < 0.01, *** *p* < 0.001, compared with the model group.).

Among viability assays that depend on the conversion of substrate to a chromogenic product by live cells, the MTT assay is still among the most versatile and popular assays [48]. We set up the effects of quercetin and kaempferol at different concentrations on the survival rate of Sertoli cells under heat stress and compared them with the control group and heat stress group (Figure 3A). The results showed that the heat stress model had a greater degree of inhibition on cell viability, and we found that the two most critical concentrations of quercetin and kaempferol on the viability of Sertoli cells were 0.1μM and 1μM. Therefore, in the follow-up study, we mainly set these two concentration gradients as the treatment group. Under high temperatures, cells first prevent polypeptide chain degeneration and false aggregation by rapidly increasing the expression of HSP70 to improve cell stress tolerance to resist apoptosis. At the same time, heat stress causes testicular SCs to rapidly accumulate a large amount of ROS. In normal testes, ROS are maintained at an acceptable level due to a balance with antioxidants [49,50,51]. The production of ROS is important for the apoptosis of germ cells and DNA damage [52]. ROS are molecules with at least one unpaired electron, making them highly unstable and extremely reactive for lipids, amino acids, and nucleic acids [53]. A 42 °Cheat treatment generates oxidative stress in the testis by upregulating ROS and downregulating the ROS/antioxidant balance, resulting in oxidative stress followed by apoptosis [54].

**Figure 4:**
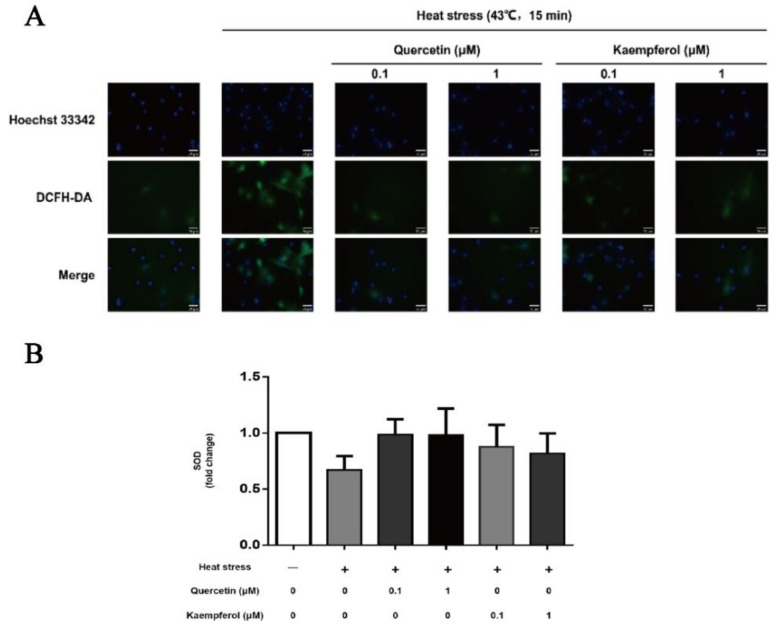
**Effects of quercetin and kaempferol on the expression of ROS and SOD in Sertoli cells induced by heat stress** (DCFH-DA was used to detect the changes of ROS content in Sertoli cells (×200) (**A**), changes in SOD activity of Sertoli cells after dry preheating stress treatment with quercetin and kaempferol (**B**). Data are shown as mean ±SEM (*n* = 3 per group.).

The present results showed that heat stimulation led to the increase in HSP70 and ROS as well as a decrease in SOD in SCs (Figure 3B and Figure 4), which was consistent with the previous literature. In addition, the present study demonstrated that treatment with 0.1 μM quercetin or 0.1 μM kaempferol reduced the expression of HSP70 and ROS in SCs as well as improved the level of SOD, thereby avoiding the cytotoxicity caused by HSP70 overexpression and helping cells to readjust the balance of oxidative stress. With increased heat stress, the oxidative balance in the testis will eventually be destroyed, and the body will also lose its regulatory ability, resulting in the destruction of testicular tissue structure and function [55]. DAPI is a common fluorescent dye used to label the nucleus. It can combine with double-stranded DNA in the nucleus and is often used to label the nucleus of fixed cells [56,57]. Hoechst 33342 is also a common fluorescent dye used to label the nucleus. It selectively combines with double-stranded DNA in the nucleus and is commonly used to label the nucleus of living cells [58,59]. Previous studies have shown that DAPI dye destroys the storage of ROS in cells when labeling nuclei, which interferes with the experimental results. Hoechst 33342 dye can not only label nuclei quickly, but also does not affect the ROS level produced in cells. Therefore, we used Hoechst 33,342 dye to mark the nucleus in the fluorescence detection of ROS, because it does not lead to a significant decrease in ROS levels in cells after labeling nuclei. ROS is of great significance in maintaining the homeostasis of the body’s physiological environment. Accumulation of excess ROS causes oxidative stress and cell apoptosis [60]. SOD is an important scavenger of ROS, which prevents tissue damage caused by free radical reaction [44]. Mild heat stress induces upregulation of SOD, indicating a robust oxidative stress response [2]. The above results showed that quercetin and kaempferol suppressed the heat-induced oxidative stress damage of testicular SCs, thereby protecting SCs, which was consistent with the results of the network pharmacological prediction.

In the present study, quercetin and kaempferol protected testicular SCs from oxidative stress injury and improved their resistance to heat stress, which was consistent with previous studies [61,62]. Quercetin and kaempferol have a weak ability to improve SOD activity of heat-damaged supporting cells, but for flavonoid monomers, their antioxidant ability lies not only in improving the level of antioxidants, such as SOD but also in their unique active structure to prevent and eliminate oxygen free radicals [63,64]. Quercetin has one more ortho phenolic hydroxyl than kaempferol. According to the structure-activity relationship theory, ortho phenolic hydroxyl improves the activity and antioxidant activity of quercetin [65]. The phenolic hydroxyl group on the benzene ring of flavonoids reacts with free radicals to form more stable semiquinone free radicals, which terminates the oxidation chain reaction. The 3-hydroxy-4-carbonyl or 4-carbonyl-5-hydroxy structure combines with the metal ion chelation that induces oxidation to inhibit the activity of metalloenzymes [66]. In addition, flavonoids also play an antioxidant role by selectively binding oxidase, changing the structure of oxidase, and inhibiting the activity of variable oxidase, and they play an anti-inflammatory role by binding with receptor proteins on cells [67,68,69], which may explain why quercetin and kaempferol effectively reduce the content of ROS with no obvious change in SOD activity. In addition, quercetin inhibits NOS activity, reduces the production of nitrite ions, reduces the production of nitric oxide superoxide anion, and increases the production of SOD, thereby resisting oxidative stress damage. Kaempferol also has a strong antioxidant capacity [70], which improves the semen quality of benzopyrene (an environmental toxin)-induced sterile mice by improving the activity of the GSH-PX antioxidant enzyme, enhancing SOD activity, and reducing cytochrome P450 (CYP450) enzyme activity [71].

**Figure 5:**
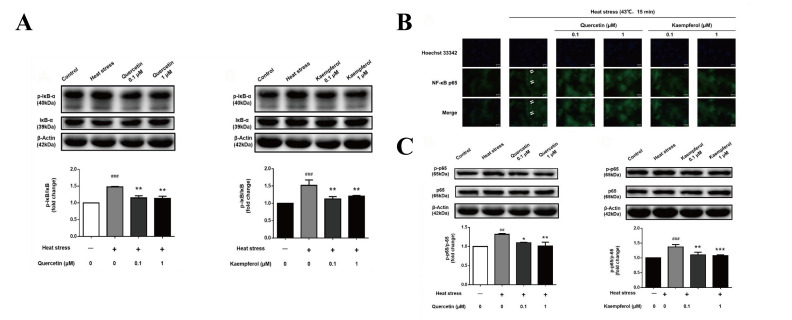
Effects of quercetin and kaempferol on the expression of p-IκB, p-NFκB-p65, and NF κB-p65 nuclear translocation in Sertoli cells after heat stress (p-IκBα expression in Sertoli cell by Western blotting (**A**), Immunofluorescence detection of NF-κB p65 nuclear translocation changes in Sertoli cells (×200) (**B**), p-NFκB expression in Sertoli cell by Western blotting (**C**). Data are shown as mean ±SEM (*n* = 3 per group.). (## *p*< 0.01, ### *p* < 0.001, compared with the control group; * *p* < 0.05, ** *p* < 0.01, *** *p* < 0.001, compared with the model group.).

When SCs suffer from excessive oxidative stress induced by heat stress, overexpression of heat shock proteins, as agonists, also activates the NF-κB signaling pathway [35], which induces cells to release a large number of cytokines, resulting in abnormal cell function [61,72,73]. Inflammation and oxidative stress cycle activation form a vicious cycle. Studies have shown that antioxidants, such as vitamin C and melatonin, [49] as well as traditional Chinese medicine or proprietary Chinese medicine, such as purslane and compound Huoxiang oral liquid, reduce and delay the increase in HSP70 caused by heat stress [33,74]. Studies have shown that anti-inflammatory drugs inhibit NF-κB in SCs, which prevents the secretion of factors to protect spermatogenic cells rather than acting directly on spermatogenic cells [75]. In addition, inflammatory factors, such as lipopolysaccharide, also activate the NF-κB signaling pathway, leading to sperm cell damage and even infertility [76,77]. In the present study, immunofluorescence analysis demonstrated that the nuclear translocation of NF-κB-p65 increased in SCs in the heat stress group (Figure 5B). In addition, the present study demonstrated that quercetin and kaempferol significantly inhibited p-IκBα and activation of the p-NF-κB-p65 signaling pathway (Figure 5A,C), thereby confirming the network pharmacology prediction results. Previous cell experiments have demonstrated that quercetin regulates TNF-α and inhibits the expression of inflammatory cytokines and proinflammatory mediators, such as IL-6, NO, COX-2, and IL-1β. Quercetin also inhibits the formation of the TLR4/MyD88/PI3K copolymer, thereby regulating the NF-κB pathway [31,78,79]. Quercetin participates in the maintenance of the dynamic balance of the levels of follicle-stimulating hormone, luteinizing hormone, and testosterone to improve the changes of seminiferous tubule epithelial cells, spermatogenic function damage, and testicular function damage caused by oxidative stress and inflammatory reaction in testicular torsion rats [80]. Kaempferol inhibits IκB kinase activation in mast cells to inhibit the NF-κB signaling pathway and downregulate the release of inflammatory mediators, such as histamine, IL-6, IL-8, IL-1β, and TNF-α [81].

The BTB, which is mainly composed of SCs, is an important protective barrier for the male reproductive system to resist external stimulation. The structural integrity of the BTB is important for germ cells to avoid the killing of the autoimmune system during spermatogenesis, to avoid harmful substances from entering the seminiferous tubules, and transport nutrients [82,83]. In addition, the adjacent SCs form tight junctions, gap junctions, and anchoring junctions, which constitute the structural basis of the BTB. The formed immune exemption zone not only protects sperm from the invasion of autoimmune reaction but also blocks the damage of pathogenic microorganisms and antigens, providing a necessary basic environment for spermatogenesis [84,85,86]. Testicular SCs are the only somatic cells in the seminiferous tubules that directly connect with germ cells, and they play an important role in the spermatogenic cycle. Testicular SCs are the upstream “nanny cells” of germ cells, which receive and integrate hormone signals, regulate spermatogenesis, and provide structural support and nutritional supply for germ cells [87]. SCs play a vital role in maintaining the homeostasis of spermatogenesis. Abnormal SC number and impaired SC physiological function directly affect the quality of sperm and even lead to germ cell apoptosis [88,89,90].

**Figure 6:**
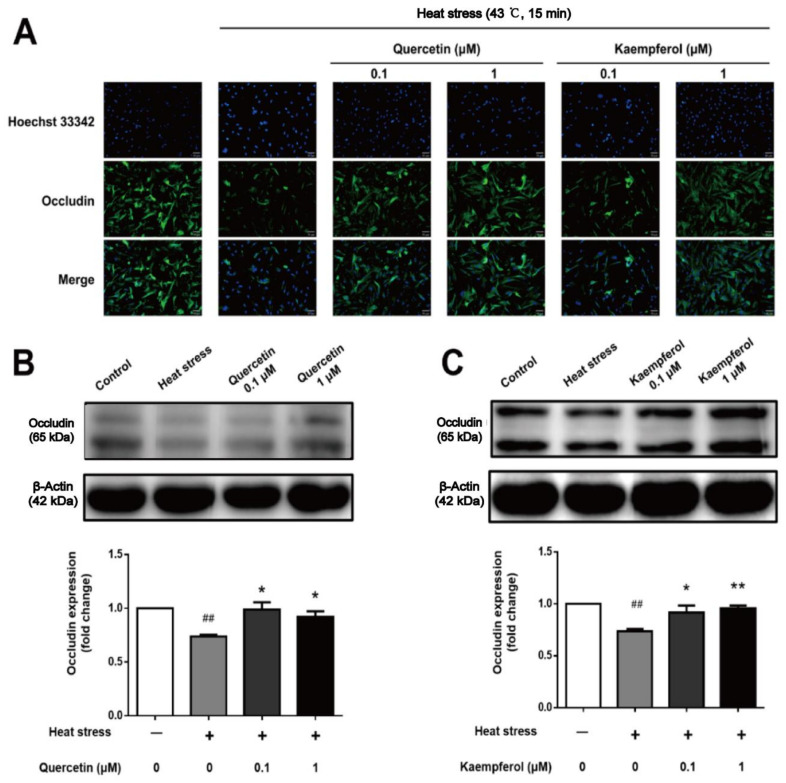
Effects of quercetin and kaempferol on the expression and localization of occludin in Sertoli cells induced by heat stress (Localization of occludin in Sertoli cells detected by immunofluorescence (×200) (**A**), Analysis of quercetin (**B**), and kaempferol (**C**), on occludin expression in Sertoli cell by Western blotting. (## *p* < 0.01, compared with the control group; * *p* < 0.05, ** *p* < 0.01, compared with the model group.).

**Figure 7:**
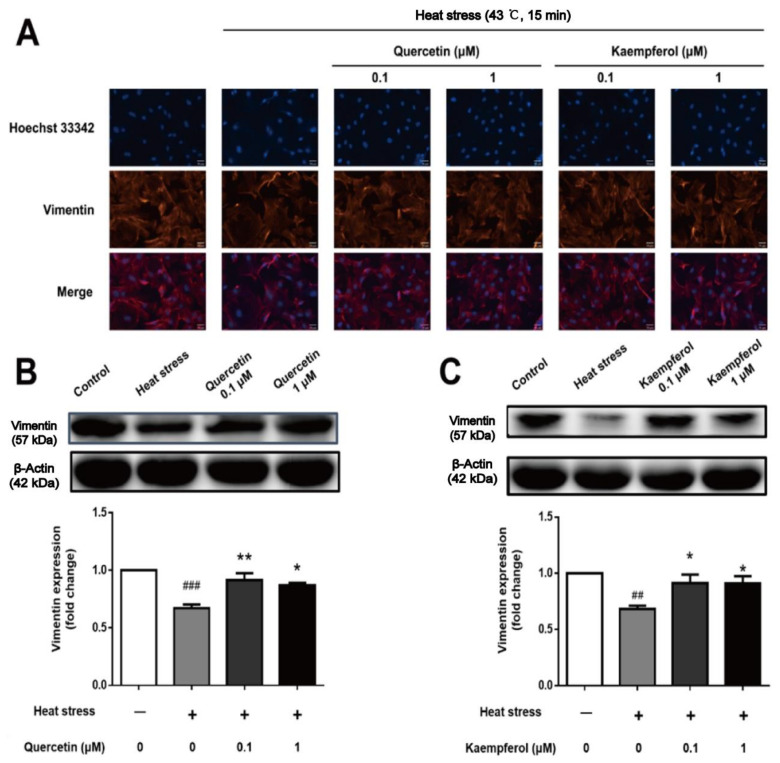
Effects of quercetin and kaempferol on the expression and localization of vimentin in Sertoli cells induced by heat stress (Localization of vimentin in Sertoli cells detected by immunofluorescence (×200) (**A**), Analysis of quercetin (**B**), and kaempferol (**C**), on vimentin expression in Sertoli cell by Western blotting (## *p* < 0.01, (### *p* < 0.01, compared with the control group; * *p* < 0.05, ** *p* < 0.01, compared with the model group.).

**Figure 8:**
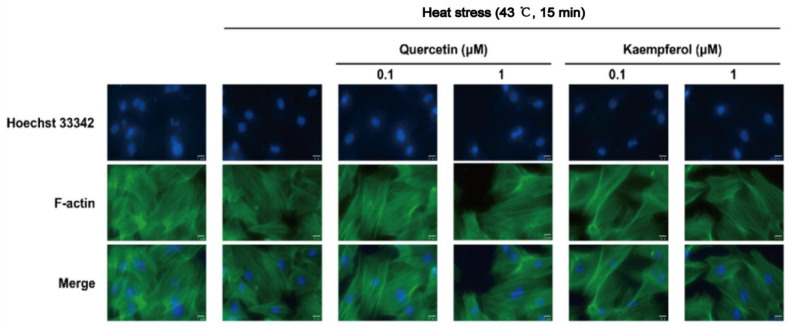
Effects of quercetin and kaempferol on the localization of F-actin in Sertoli cells induced by heat stress (Localization of F-actin in Sertoli cells detected by immunofluorescence (×200)).

Our results confirmed that quercetin and kaempferol have protective effects via upregulating expression and mediating distribution of the following main components of the BTB after heat stress treatment: occludin, a supporting cell tight junction protein; vimentin, a cytoskeleton component composed of intermediate filaments; and F-actin, a microfilament component (Figure 6, Figure 7 and Figure 8). Interestingly, the F-actin damage in the supporting cytoskeleton induced by heat stress is similar to that caused by an inflammatory reaction, that is, NF-κB regulates the expression of the IL-1α cytokine by inducing Eps8 to separate from the surface of SC, which destroys the F-actin bundle and cell connection, resulting in SC membrane internalization and ultimately BTB injury [91,92,93,94,95]. In contrast, the vimentin structure damage and expression of vimentin in SCs induced by heat stress are consistent with the decrease and abnormal distribution of vimentin expression in the cadmium-induced testicular SC stress model [96]. These findings suggest that occludin, vimentin, and F-actin in SCs all significantly contribute to the normal function of the BTB. It is well known that the BTB plays an important role in maintaining spermatogenesis, and the damage and recovery of spermatogenesis are closely related to the disintegration and recovery of these tight junction proteins and intermediate filaments. The adverse effect of decreased expression of structural proteins associated with various testicular injury models is due to the collapse of structural protein filaments of supporting cells from the cell membrane, which may lead to the separation of spermatogenic cells, causing spermatogenic cells to undergo apoptosis due to the loss of support and nutrition provided by SCs. The regulation of these proteins may be an important mechanism target for the overall protective effect of these two compounds on supporting cells, which may supplement the lack of predicted network pharmacological targets to a certain extent.

## 4. Materials and Methods

### 4.1. Online Databases and Software

Network pharmacology was used to investigate the pharmacological mechanisms of quercetin and kaempferol against spermatogenesis disorders. The target genes of quercetin, kaempferol, and spermatogenesis disorders were collected through online databases. Annotation and enrichment were performed using online tools. The following databases and software were used in this study: BindingDB database (bindingdb.org) (accessed on 6 February 2019) [97]; Traditional Chinese Medicine systems pharmacology database and analysis platform (tcmspw.com/tcmsp.php) (accessed on 6 February 2019) [98]; TCMSP database (Tcm.cmu.edu.tw); DrugBank database (www.drugbank.ca) (accessed on 6 February 2019) [99]; UniProt database (www.uniprot.org) (accessed on 10 February 2019) [100]; Online Mendelian Inheritance in Man (OMIM) database (www.omim.org) (accessed on 10 February 2019) [101]; GeneCards database (www.genecards.org) (accessed on 15 February 2019) [102]; Venn Diagrams (bioinformatics.psb.ugent.be/webtools/Venn/) (accessed on 4 March 2019); Metascape (Metascape.org) (accessed on 4 March 2019) [103]; VarElect (ve.genecards.org) (accessed on 6 March 2019) [104]; STRING database (string-db.org) (accessed on 6 March 2019); and Cytoscape software (3.6.1).

The “quercetin” and “kaempferol” search terms were used to search the DrugBank database, and the 3D structures and SMILES number of the two compounds were collected for target prediction. Based on the target information of the two compounds in the BindingDB, TCMSP, ChEMBL, and STITCH databases, the UniProt database was searched to identify the human target and record its gene name. The network diagrams of the quercetin and kaempferol targets were constructed by Cytoscape software. The intersection of quercetin and kaempferol targets with spermatogenesis disorders was analyzed by a Wayne diagram. Quercetin and kaempferol were considered potential target genes for the treatment of spermatogenesis disorders.

### 4.2. Materials and Reagents

Quercetin (S2391) and kaempferol (S2314) were purchased from Selleck (Houston, TX, USA). RIPA lysis buffer, proteinase inhibitor cocktail, DMSO, and the total SOD activity detection kit (WST-8) were purchased from Beyotime (Shanghai, China). Hoechst 33342 was purchased from Sigma (St. Louis, MI, USA). Dichlorodihydrofluorescein acetoacetate (DCFH-DA) was purchased from Solarbio (Beijing, China). The BCA protein assay was purchased from Thermo Fisher (Waltham, MA, USA).

### 4.3. Cell Isolation, Culture, and Heat Stress Procedure

SCs were isolated from adult rat testes using a two-step enzymatic digestion with collagenase IV and trypsin, which is a common method of isolating SCs with a purity exceeding 95%. The SCs were cultured at 37 °C in a 5% CO_2_ humidified incubator and then randomly allocated to the control group, heat stress group (model), quercetin treatment group, and kaempferol treatment group in triplicate. SCs were placed in a 43 °C thermostatically controlled water bath for 15 min in a sealing pocket for heat stress treatment.

### 4.4. Cell Proliferation Assay

For the MTT assay to evaluate cell proliferation, SCs were seeded into 96-well plates, and 200 µL of culture medium was added to each well. SCs were randomly divided into different treatments. After treatment for 24 h, 100 µL of culture medium was removed, and 15 µL of MTT solution (5 g/L) was added to each well followed by additional culture for 4 h. Finally, the culture medium was discarded, and 150 µL of DMSO was added. After the mixture was fully reacted in the dark on a shaker for 15 min, the absorbance was measured at 570 nm.

### 4.5. Determination of ROS by DCFH-DA

SCs were seeded into 96-well plates (2 × 104 cells/mL in 200 μL/well) and cultured to allowed adherence. Cell morphology was observed with an inverted microscope. The control group and model group were treated with low serum medium (1% FBS + DMEM/F12 medium) for 24 h, and the quercetin and kaempferol groups were treated with the corresponding treatment for 24 h. After 24 h, 100 μL of DCFH-DA (prepared with PBS) was added to each well followed by incubation for 20 min at 3 °C, and 10 μL of 10× Hoechst 33,342 was added to each well followed by incubation at 3 °C for 10 min after mixing. The ROS production level in SCs of testis was observed under a fluorescence microscope. Three random fields were selected in each group for imaging.

### 4.6. SOD Activity Assay

SC intracellular SOD activity was measured with a total SOD activity detection kit (WST-8) according to the manufacturer’s instructions. After drug treatment, cells were washed once with PBS and collected by centrifugation. SCs were fully lysed in SOD sample preparation solution, and the supernatants were collected by centrifugation at 12,000× *g* at 4 °C for 5 min. The absorbance was detected at 450 nm with a multimode microplate reader, and the SOD activity was calculated.

### 4.7. Western Blots

SCs treated by heat stress, quercetin, or kaempferol were harvested and immediately lysed with RIPA lysis buffer containing a proteinase inhibitor cocktail. The total proteins were collected, and the concentrations were determined by a BCA protein assay. Equal amounts of protein were loaded and separated by 10% SDS-PAGE gels, and the proteins were then transferred onto Millipore polyvinylidene fluoride (PVDF) membranes and blocked with Tris-buffered saline with 0.1% Tween-20 (TBS-T) containing 5% skim milk for 2 h. The membranes were then incubated with primary antibodies, including anti-HSP70, anti-p-IκB, anti-IκB, anti-p-NFκB-p65, anti-NFκB-p65 (Cell Signaling, USA), anti-β-actin, anti-vimentin, and anti-occludin (Proteintech, Wuhan, China) antibodies, at 4 °C overnight. IκB, NFκB-p65, and β-actin expression levels were used as the controls. Antibodies were detected using HRP-conjugated secondary antibodies (Thermo Scientific, Waltham, MA, USA).

### 4.8. Immunofluorescence Staining

SCs were seeded onto slides in 12-well culture plates. After treatment, cells were fixed with ice-cold methanol for 5 min, blocked with 3% bovine serum albumin (BSA) for 30 min, and incubated with primary anti-NFκB-p65, occludin, and vimentin (1:200) antibodies at 4 °C overnight. The slides were then incubated with Alexa Fluor^®^ 488 (Abcam, USA) (1:200) or FITC-phalloidin (Solarbio, USA) (1:400 and no antibody incubation on the first day) at 37 °C for 2 h followed by incubation with DAPI (Solarbio, USA) for 10 min. Fluorescence was observed with a fluorescent inverted microscope (Olympus, Shinjuku, Japan).

### 4.9. Statistical Analysis

Statistical analyses were performed with GraphPad Prism 6 software (USA). Data are presented as the mean ± standard error of the mean (SEM), and data were evaluated by two-tailed unpaired Student’s *t*-test and one-way ANOVA. Results were considered statistically significant at *p* < 0.05.

## 5. Conclusions

**Figure 9:**
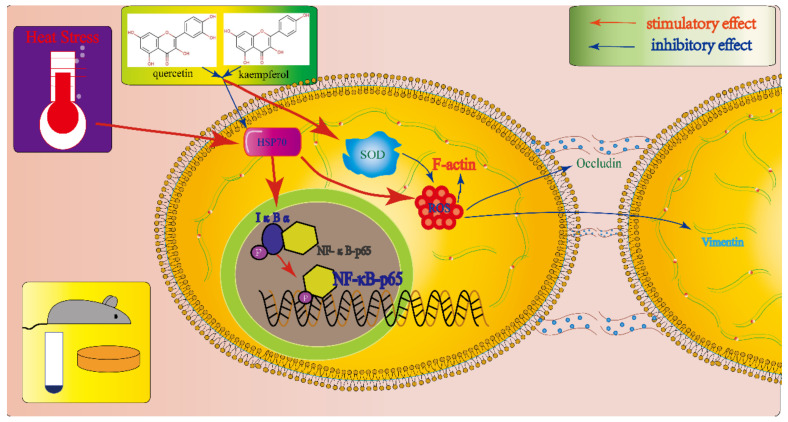
Proposed mechanisms of quercetin and kaempferol in the treatment of Sertoli cell injury induced by heat stress.

The results of this study show that Sertoli cells are very sensitive to heat stress, which is manifested in the destruction of the balance of oxidative stress caused by the increase in HSP70 expression, the increase in ROS and the decrease in SOD activity, the rise of the level of p-IκB and p-NFκB p65 and the abnormal changes in the content and location of occludin, vimentin and F-actin. In general, these findings suggest that quercetin and kaempferol may play a role in resisting heat stress-induced SCs injury through antioxidant stress and anti-inflammatory (Figure 9). This indicates that flavonoids represented by quercetin and kaempferol may play a role in the prevention and treatment of spermatogenic disorders through antioxidant stress, anti-inflammatory, maintenance of cytoskeleton, and tight junctions between cells.

## Data Availability

Restrictions apply to the availability of these data. Data was obtained from third party and are available at BindingDB database (bindingdb.org); Traditional Chinese Medicine systems pharmacology database and analysis platform (tcmspw.com/tcmsp.php); TCMSP database (Tcm.cmu.edu.tw); DrugBank database (www.drugbank.ca); UniProt database (www.uniprot.org); Online Mendelian Inheritance in Man (OMIM) database (www.omim.org); GeneCards database (www.genecards.org) with the permission of third party. Data on experimental validation can be obtained in the article.

## Supplementary Materials

The following supporting information can be downloaded at: https://www.mdpi.com/article/10.3390/ijms231911163/s1.
