# Supplementary material for: Probing the Potential Mechanism of Quercetin and Kaempferol against Heat Stress-Induced Sertoli Cell Injury: Through Integrating Network Pharmacology and Experimental Validation
Source: Int J Mol Sci. 2022 Sep 22;23(19):11163. doi: 10.3390/ijms231911163 (PMC9570440; doi:10.3390/ijms231911163)
Supplement: Supplementary file 1 [file ijms-23-11163-s001.zip › additional files CWS_Editorial_Certificate.pdf]

Charlesworth Author Services

EDITORIAL CERTIFICATE

This document certifies that the manuscript below was edited for correct English language usage, grammar, punctuation and spelling by qualified native English speaking editors at Charlesworth Author Services.

Paper Title:

Probing the potential mechanism of flavonoids quercetin and kaempferol against Heat stress-induced Sertoli cell injury: Through Integrating Network Pharmacology and Experimental Validation

Author:

殿龙 刘

Date certificate issued:

August 19, 2022

cwauthors.com
