# Supplementary material for: Probing the Potential Mechanism of Quercetin and Kaempferol against Heat Stress-Induced Sertoli Cell Injury: Through Integrating Network Pharmacology and Experimental Validation
Source: Int J Mol Sci. 2022 Sep 22;23(19):11163. doi: 10.3390/ijms231911163 (PMC9570440; doi:10.3390/ijms231911163)
Supplement: Supplementary file 1 [file ijms-23-11163-s001.zip › additional files targets of quercetin and kaempferol.pdf]

Supplementary materials

Table S1. Quercetin target list

Gene	Abbreviation	Database
ATP-binding cassette sub-family G member 2	ABCA2	TCMSP、Bindding DB
P-glycoprotein 1	ABCB1	Bindding DB
Canalicular multispecific organic anion transporter 1	ABCC2	CHEMBL
ATP-binding cassette sub-family G member 2	ABCG2	CHEMBL
Acetyl-CoA carboxylase 1	ACACA	TCMSP
Acetylcholinesterase	ACHE	TCMSP、CHEMBL
Ecto-5'-nucleotidase (e5'NT)	ACPP	TCMSP、Bindding DB
Adenosine receptor A1	ADORA1	Bindding DB
Adenosine Receptors A2a (A2a)	ADORA2A	Bindding DB
Adenosine receptor A3	ADORA3	Bindding DB
Beta-2 adrenergic receptor	ADRB2	TCMSP
Aryl hydrocarbon receptor	AHR	TCMSP、Bindding DB
Activator of 90 kDa heat shock protein ATPase homolog 1	AHSA1	TCMSP
Aldose reductase	AKR1B1	TCMSP、Bindding DB、CHEMBL
RAC-alpha serine/threonine-protein kinase	AKT1	TCMSP
Retinal dehydrogenase 1	ALDH1A1	CHEMBL
ALK tyrosine kinase receptor	ALK	CHEMBL
Arachidonate 12-lipoxygenase, 12S-type	ALOX12	CHEMBL、Bindding DB
Arachidonate 15-lipoxygenase	ALOX15	Bindding DB
Arachidonate 5-lipoxygenase	ALOX5	TCMSP、Bindding DB、CHEMBL
Alpha-amylase 1	AMY1A	CHEMBL
Androgen receptor	AR	TCMSP
ATPase family AAA domain-containing protein 5	ATAD5	CHEMBL
ATP synthase subunit alpha, mitochondrial	ATP5F1A	STITCH
ATP synthase subunit beta, mitochondrial	ATP5F1B	STITCH
ATP synthase subunit gamma, mitochondrial	ATP5F1C	STITCH
Aurora kinase B/Inner centromere protein	AURKB	Bindding DB
Vasopressin V2 receptor	AVPR2	CHEMBL
Apoptosis regulator BAX	BAX	TCMSP、STITCH
Cholinesterase	BCHE	CHEMBL
Apoptosis regulator Bcl-2	BCL2	TCMSP
Bcl-2-like protein 1	BCL2L1	TCMSP

Table S1 Quercetin target list (Continued)

Gene	Abbreviation	Database
Baculoviral IAP repeat-containing protein 5	BIRC5	TCMSP

Bloom syndrome protein	BLM	CHEMBL
Carbonic anhydrase 1	CA1	CHEMBL
Carbonic anhydrase 12	CA12	Bindding DB
Carbonic anhydrase 14	CA14	CHEMBL
Carbonic anhydrase 2	CA2	Bindding DB、CHEMBL
Carbonic anhydrase 3	CA3	CHEMBL
Carbonic anhydrase 4	CA4	Bindding DB
Carbonic anhydrase 5A, mitochondrial	CA5A	CHEMBL
Carbonic anhydrase 6	CA6	CHEMBL
Carbonic anhydrase 7	CA7	Bindding DB、CHEMBL
Calcium/calmodulin-dependent protein kinase type II subunit beta	CAMK2B	CHEMBL
Calcium/calmodulin-dependent protein kinase type II subunit delta	CAMK2D	CHEMBL
Calcium/calmodulin-dependent protein kinase type IV	CAMK4	CHEMBL
Caspase-3	CASP5	TCMSP
Caspase-8	CASP8	TCMSP
Caspase-9	CASP9	TCMSP
Caveolin-1	CAV1	TCMSP
Carbonyl reductase [NADPH] 3	CBR3	CHEMBL
C-C motif chemokine 2	CCL2	TCMSP
G2/mitotic-specific cyclin-B1	CCNB1	TCMSP
G1/S-specific cyclin-D1	CCND1	TCMSP
CD40 ligand	CD40LG	TCMSP
Cell division control protein 2 homolog	CDK1	TCMSP、Bindding DB、CHEMBL
Cyclin-Dependent Kinase 5 (CDK5)	CDK5	Bindding DB
Cyclin-Dependent Kinase 6 (CDK6)	CDK6	Bindding DB
Cyclin-dependent kinase inhibitor 1	CDKN1A	TCMSP
Cyclin-dependent kinase inhibitor 2A, isoforms 1/2/3	CDKN2A	TCMSP
Serine/threonine-protein kinase Chk2	CHEK2	TCMSP、CHEMBL

Table S1 Quercetin target list (Continued)

Gene	Abbreviation	Database
Inhibitor of nuclear factor kappa-B kinase subunit alpha	CHUK	TCMSP
Claudin-4	CLDN4	TCMSP
Dual specificity protein kinase CLK1	CLK1	CHEMBL
Dual specificity protein kinase CLK3	CLK3	CHEMBL
Collagen alpha-1(I) chain	COL1A1	TCMSP
Collagen alpha-1(III) chain	COL3A1	TCMSP
C-reactive protein	CRP	TCMSP

Casein kinase I isoform gamma-1	CSNK1G1	CHEMBL
Casein kinase I isoform gamma-2	CSNK1G2	CHEMBL
Casein kinase I isoform gamma-3	CSNK1G3	CHEMBL
Cathepsin D	CTSD	TCMSP
Cathepsin L1	CTSL	CHEMBL
C-X-C motif chemokine 10	CXCL10	TCMSP
C-X-C motif chemokine 11	CXCL11	TCMSP
C-X-C motif chemokine 2	CXCL2	TCMSP
Interleukin-8	CXCL8	TCMSP
C-X-C chemokine receptor type 1	CXCR1	CHEMBL
Aromatase (CYP19)	CYP19A1	Bindding DB、CHEMBL
Cytochrome P450 1A1	CYP1A1	TCMSP、Bindding DB、STITCH
Cytochrome P450 1A2	CYP1A2	TCMSP、Bindding DB、CHEMBL
Cytochrome P450 1B1	CYP1B1	TCMSP、Bindding DB、STITCH
Cytochrome P450 2C8	CYP2C8	CHEMBL、STITCH
Cytochrome P450 2C9	CYP2C9	CHEMBL
Cytochrome P450 3A4	CYP3A4	TCMSP
Death-associated protein kinase 1	DAPK1	CHEMBL
DDB1- and CUL4-associated factor 5	DCAF5	TCMSP
Type I iodothyronine deiodinase	DIO1	TCMSP
Myotonin-protein kinase	DMPK	CHEMBL
Dipeptidyl peptidase IV	DPP4	TCMSP、CHEMBL
Dual oxidase 2	DUOX2	TCMSP
Transcription factor E2F1	E2F1	TCMSP

Table S1 Quercetin target list (Continued)

Gene	Abbreviation	Database
Transcription factor E2F2	E2F2	TCMSP
Pro-epidermal growth factor	EGF	TCMSP
MER intracellular domain/EGFR extracellular domain chimera	EGFR	Bindding DB、CHEMBL
Histone-lysine N-methyltransferase EHMT2	EHMT2	CHEMBL
Eukaryotic translation initiation factor 6	EIF6	TCMSP
ETS domain-containing protein Elk-1	ELK1	TCMSP
Receptor tyrosine-protein kinase erbB-2	ERBB2	TCMSP
Receptor tyrosine-protein kinase erbB-3	ERBB3	TCMSP
Estrogen receptor	ESR1	CHEMBL
Thrombin	F2	TCMSP、Bindding DB
Tissue factor	F3	TCMSP
Coagulation factor VII	F7	TCMSP
Enoyl-acyl-carrier protein reductase	FASN	Bindding DB

Tyrosine-protein kinase receptor FLT3	FLT3	Bindding DB、CHEMBL
Proto-oncogene c-Fos	FOS	TCMSP
Gamma-aminobutyric acid receptor subunit alpha-1	GABRA1	TCMSP
AXL receptor tyrosine kinase	GAS6	Bindding DB
Gap junction alpha-1 protein	GJA1	TCMSP
Glyoxalase 1 (GLO1)	GLO1	Bindding DB、CHEMBL
Glycogen Synthase Kinase-3, beta	GSK3B	Bindding DB
Glutathione reductase, mitochondrial	GSR	CHEMBL
Glutathione S-transferase Mu 1	GSTM1	TCMSP
Glutathione S-transferase Mu 2	GSTM2	TCMSP
Glutathione S-transferase P	GSTP1	TCMSP
Hyaluronan synthase 2	HAS2	TCMSP
Tyrosine-protein kinase HCK	HCK	STITCH
3-hydroxyisobutyryl-CoA hydrolase, mitochondrial	HIBCH	STITCH
Hypoxia-inducible factor 1-alpha	HIF1A	TCMSP
Hexokinase-2	HK2	TCMSP
Heme oxygenase 1	HMOX1	TCMSP
Corticosteroid 11-beta-dehydrogenase isozyme 1	HSD11B1	CHEMBL
Estradiol 17-beta-dehydrogenase 1	HSD17B1	CHEMBL
3-hydroxyacyl-CoA dehydrogenase type-2	HSD17B10	CHEMBL
Estradiol 17-beta-dehydrogenase 2	HSD17B2	Bindding DB、CHEMBL

Table S1 Quercetin target list (Continued)

Gene	Abbreviation	Database
Heat shock factor protein 1	HSF1	TCMSP
Heat shock protein HSP 90	HSP90AA1	TCMSP
78 kDa glucose-regulated protein	HSPA5	TCMSP
Heat shock protein beta-1	HSPB1	TCMSP、CHEMBL
Intercellular adhesion molecule 1	ICAM1	TCMSP
Isocitrate dehydrogenase [NADP] cytoplasmic	IDH1	CHEMBL
Interferon gamma	IFNG	TCMSP
Insulin-like growth factor I receptor	IGF1R	Bindding DB
Insulin-like growth factor II	IGF2	TCMSP
Insulin-like growth factor-binding protein 3	IGFBP3	TCMSP
Interleukin-10	IL10	TCMSP
Interleukin-1 alpha	IL1A	TCMSP
Interleukin-1 beta	IL1B	TCMSP
Interleukin-2	IL2	TCMSP
Interleukin-6	IL6	TCMSP
Insulin receptor	INSR	TCMSP
Interferon regulatory factor 1	IRF1	TCMSP
Tyrosine-protein kinase JAK1	JAK1	CHEMBL
Transcription factor AP-1	JUN	TCMSP

Potassium voltage-gated channel subfamily H member 2	KCNH2	TCMSP
Vascular endothelial growth factor receptor 2	KDR	CHEMBL
Interstitial collagenase	LSS	TCMSP
Monoamine oxidase	MAOA	Bindding DB
Amine oxidase [flavin-containing] B	MAOB	TCMSP、CHEMBL
Dual specificity mitogen-activated protein kinase kinase 6	MAP2K6	CHEMBL
Mitogen-activated protein kinase kinase kinase 5	MAP3K5	CHEMBL
Mitogen-activated protein kinase 1	MAPK1	TCMSP
MAP kinase-activated protein kinase 2	MAPKAPK2	CHEMBL
MAP kinase-activated protein kinase 5	MAPKAPK5	CHEMBL
Induced myeloid leukemia cell differentiation protein Mcl-1	MCL1	STITCH
Hepatocyte growth factor receptor	MET	Bindding DB
Maltase-glucoamylase, intestinal	MGAM	TCMSP

Table S1 Quercetin target list (Continued)

Gene	Abbreviation	Database
Matrix metalloproteinase-12 (MMP12)	MMP12	Bindding DB
72 kDa type IV collagenase	MMP2	TCMSP
Stromelysin-1	MMP3	TCMSP
Matrix metalloproteinase-9	MMP9	TCMSP、CHEMBL
DNA-3-methyladenine glycosylase	MPG	CHEMBL
Myeloperoxidase	MPO	TCMSP、CHEMBL
Myc proto-oncogene protein	MYC	TCMSP
Neutrophil cytosol factor 1	NCF1	TCMSP
Nuclear receptor coactivator 2	NCOA2	TCMSP
Serine/threonine-protein kinase Nek6	NEK6	CHEMBL
Sialidase	NEU1	Bindding DB
Nuclear factor erythroid 2-related factor 2	NFE2L2	TCMSP
Nuclear factor NF-kappa-B p105 subunit	NFKB1	CHEMBL
NF-kappa-B inhibitor alpha	NFKBIA	TCMSP
Homeobox protein Nkx-3.1	NKX3-1	TCMSP
Nitric oxide synthase, endothelial	NOS3	TCMSP
NADPH oxidase 4	NOX4	Bindding DB
Puromycin-sensitive aminopeptidase	NPEPPS	TCMSP
Neuropeptide S receptor	NPSR1	CHEMBL
NAD(P)H dehydrogenase [quinone] 1	NQO1	TCMSP
Nuclear receptor subfamily 1 group I member 2	NR1I2	TCMSP
Nuclear receptor subfamily 1 group I member 3	NR1I3	TCMSP
NUAK family SNF1-like kinase 1	NUAK1	CHEMBL
Ornithine decarboxylase	ODC1	TCMSP
Serine/threonine-protein kinase PAK 4	PAK4	CHEMBL

Serine/threonine-protein kinase PAK 5	PAK5	CHEMBL
Poly [ADP-ribose] polymerase 1	PARP1	TCMSP
Lymphokine-activated killer T-cell-originated protein kinase	PBK	CHEMBL
Procollagen C-endopeptidase enhancer 1	PCOLCE	TCMSP
Phosphatidylinositol-4,5-bisphosphate 3-kinase catalytic subunit, gamma isoform	PIK3CG	TCMSP、Bindding DB、CHEMBL
Serine/threonine-protein kinase PIM	PIM1	Bindding DB、STITCH
Peptidyl-prolyl cis-trans isomerase NIMA-interacting 1	PIN1	CHEMBL

Table S1 Quercetin target list (Continued)

Gene	Abbreviation	Database
Phospholipase A2	PLA2G1B	CHEMBL
Urokinase-type plasminogen activator	PLAU	TCMSP、CHEMBL
Plasminogen	PLG	CHEMBL
DNA polymerase beta	POLB	CHEMBL
Serum paraoxonase/arylesterase 1	PON1	TCMSP
NADPH--cytochrome P450 reductase	POR	TCMSP
Peroxisome proliferator-activated receptor alpha	PPARA	TCMSP
Peroxisome proliferator-activated receptor delta	PPARD	TCMSP
Peroxisome proliferator-activated receptor gamma	PPARG	TCMSP、CHEMBL
5'-AMP-activated protein kinase catalytic subunit alpha-2	PRKAA2	CHEMBL
Protein kinase C alpha type	PRKCA	TCMSP、CHEMBL
Protein kinase C beta type	PRKCB	TCMSP
Trypsin-1	PRSS1	TCMSP
lens epithelium-derived growth factor p75	PSIP1	Bindding DB
26S proteasome non-ATPase regulatory subunit 3	PSMD3	TCMSP
Phosphatidylinositol-3,4,5-trisphosphate 3-phosphatase and dual-specificity protein phosphatase PTEN	PTEN	TCMSP
Prostaglandin E2 receptor EP3 subtype	PTGER3	TCMSP
Prostaglandin G/H synthase 1	PTGS1	TCMSP、CHEMBL
Prostaglandin G/H synthase 2	PTGS2	TCMSP、Bindding DB
Glycogen phosphorylase, liver form	PYGL	CHEMBL
RAF proto-oncogene serine/threonine-protein kinase	RAF1	TCMSP
Rap guanine nucleotide exchange factor 3	RAPGEF3	CHEMBL
Ras GTPase-activating protein 1	RASA1	TCMSP
Ras association domain-containing protein 1	RASSF1	TCMSP
Retinoblastoma-associated protein	RB1	TCMSP
Transcription factor p65	RELA	TCMSP
Regulator of G-protein signaling 4	RGS4	CHEMBL
Ribosomal protein S6 kinase beta-1	RPS6KB1	CHEMBL

Protein CBFA2T1	RUNX1T1	TCMSP
Runt-related transcription factor 2	RUNX2	TCMSP
Retinoic acid receptor RXR-alpha	RXRA	TCMSP
Sodium channel protein type 5 subunit alpha	SCN5A	TCMSP

Table S1 Quercetin target list (Continued)

Gene	Abbreviation	Database
E-selectin	SELE	TCMSP
Plasminogen activator inhibitor 1	SERPINE1	TCMSP
Solute carrier family 2, facilitated glucose transporter member 2	SLC2A2	STITCH
Solute carrier family 2, facilitated glucose transporter member 4	SLC2A4	TCMSP
Solute carrier organic anion transporter family member 1B3	SLCO1B3	CHEMBL
STE20-like serine/threonine-protein kinase	SLK	CHEMBL
Mothers against decapentaplegic homolog 3	SMAD3	CHEMBL
Superoxide dismutase [Cu-Zn]	SOD1	TCMSP
Osteopontin	SPP1	TCMSP
SRC	SRC	Bindding DB、CHEMBL
Signal transducer and activator of transcription 1-alpha/beta	STAT1	TCMSP
Signal transducer and activator of transcription 6	STAT6	CHEMBL
Serine/threonine-protein kinase 17A	STK17A	CHEMBL
Serine/threonine-protein kinase 17B	STK17B	STITCH
Serine/threonine-protein kinase 25	STK25	CHEMBL
Serine/threonine-protein kinase 4	STK4	CHEMBL
Estrogen sulfotransferase	SULT1E1	TCMSP
Tyrosine-protein kinase SYK	SYK	CHEMBL
Synapsin-1	SYN1	Bindding DB
Transforming growth factor beta-1	TGFB1	TCMSP
Thrombomodulin	THBD	TCMSP
Thyroid hormone receptor beta	THRB	CHEMBL
Tumor necrosis factor	TNF	TCMSP
TRAF2 and NCK-interacting protein kinase	TNIK	CHEMBL
Human diphtheria toxin-like ADP-ribosyltransferase (ARTD5 or PARP5a)	TNKS	Bindding DB
Human diphtheria toxin-like ADP-ribosyltransferase (ARTD6 or PARP5b)	TNKS2	Bindding DB
DNA topoisomerase 1	TOP1	TCMSP、Bindding DB
DNA topoisomerase 2-alpha	TOP2A	TCMSP、CHEMBL
DNA topoisomerase II	TOP2B	TCMSP

Table S1 Quercetin target list (Continued)

Gene	Abbreviation	Database
Cellular tumor antigen p53	TP53	TCMSP、CHEMBL
TTR	TTR	Bindding DB
Tyrosinase	TYR	Bindding DB、CHEMBL
UDP-glucuronosyltransferase 1-3	UGT1A3	CHEMBL
UDP-glucuronosyltransferase 1-4	UGT1A4	CHEMBL
UDP-glucuronosyltransferase 1-9	UGT1A9	CHEMBL
Vascular cell adhesion protein 1	VCAM1	TCMSP
Vascular endothelial growth factor A	VEGFA	TCMSP
Serine/threonine-protein kinase VRK2	VRK2	CHEMBL
Inactive serine/threonine-protein kinase VRK3	VRK3	CHEMBL
Xanthine oxidase	XDH	TCMSP、Bindding DB、CHEMBL

Note: there are 262 quercetin targets in total

Table S2 Kaempferol target list

Gene	Abbreviation	Database
P-glycoprotein 1	ABCB1	Bindding DB、CHEMBL
Multidrug resistance-associated protein 1	ABCC1	CHEMBL
ATP-binding cassette sub-family G member 2	ABCG2	CHEMBL
Acetylcholinesterase	ACHE	TCMSP、CHEMBL
Ecto-5'-nucleotidase (e5'NT)	ACPP	Bindding DB
Adenosine receptor A1	ADORA1	Bindding DB
Adenosine Receptors A2a (A2a)	ADORA2A	Bindding DB
Adenosine receptor A3	ADORA3	Bindding DB
Alpha-1B adrenergic receptor	ADRA1B	TCMSP
Aryl hydrocarbon receptor	AHR	TCMSP、Bindding DB、STITCH、CHEMBL
Activator of 90 kDa heat shock protein ATPase homolog 1	AHSA1	TCMSP
Aldose reductase (AR)	AKR1B1	Bindding DB、CHEMBL
RAC-alpha serine/threonine-protein kinase	AKT1	TCMSP
Arachidonate 12-lipoxygenase	ALOX12	Bindding DB
Arachidonate 15-lipoxygenase	ALOX15	Bindding DB、CHEMBL
Arachidonate 15-lipoxygenase, type II	ALOX15B	Bindding DB
Arachidonate 5-lipoxygenase	ALOX5	TCMSP、Bindding DB、CHEMBL
Salivary alpha-amylase	AMY1A	CHEMBL
DNA-(apurinic or apyrimidinic site) lyase	APEX1	CHEMBL
Beta amyloid A4 protein	APP	CHEMBL
Androgen receptor	AR	TCMSP、CHEMBL
Aurora kinase B/Inner centromere protein	AURKB	Bindding DB
Beta-secretase 1	BACE1	CHEMBL

Apoptosis regulator BAX	BAX	TCMSP
Butyrylcholinesterase	BCHE	CHEMBL
Apoptosis regulator Bcl-2	BCL2	TCMSP
Carbonic anhydrase I	CA1	CHEMBL
Carbonic anhydrase 12	CA12	Bindding DB、CHEMBL
Carbonic anhydrase 2	CA2	Bindding DB、CHEMBL
Carbonic anhydrase 4	CA4	Bindding DB、CHEMBL
Carbonic anhydrase 7	CA7	Bindding DB、CHEMBL
Calmodulin	CALM	TCMSP

Table S2 Kaempferol target list (Continued)

Gene	Abbreviation	Database
Caspase-3	CASP6	TCMSP
Catalase	CAT	CHEMBL
Carbonyl reductase [NADPH] 1	CBR1	Bindding DB
Cell division control protein 2 homolog	CDK1	TCMSP、Bindding DB、STITCH
Cyclin-Dependent Kinase 5	CDK5	Bindding DB
Cyclin-Dependent Kinase 6	CDK6	Bindding DB、CHEMBL
CFTR	CFTR	Bindding DB
Muscarinic acetylcholine receptor M1	CHRM1	TCMSP
Muscarinic acetylcholine receptor M2	CHRM2	TCMSP
CDGSH iron-sulfur domain-containing protein 1	CISD1	CHEMBL
Casein Kinase II	CSNK2A1	Bindding DB、CHEMBL
Carboxy-terminal domain RNA polymerase II polypeptide A small phosphatase 1	CTDSP1	CHEMBL
Aromatase (CYP19)	CYP19A1	Bindding DB
Cytochrome P450 1A1	CYP1A1	TCMSP、Bindding DB、CHEMBL
Cytochrome P450 1A	CYP1A2	TCMSP、Bindding DB、CHEMBL
Cytochrome P450 1B1	CYP1B1	TCMSP、Bindding DB、STITCH、CHEMBL
Cytochrome P450 2C19	CYP2C19	CHEMBL
Cytochrome P450 2C9	CYP2C9	CHEMBL
Cytochrome P450 2D6	CYP2D6	CHEMBL
Cytochrome P450 3A4	CYP3A4	TCMSP、CHEMBL
Death-associated protein kinase 1	DAPK1	CHEMBL
Type I iodothyronine deiodinase	DIO1	TCMSP
Dipeptidyl peptidase IV	DPP4	TCMSP、CHEMBL
MER intracellular domain/EGFR extracellular domain chimera	EGFR	Bindding DB、CHEMBL
Estrogen receptor alpha	ESR1	CHEMBL

Estradiol receptor beta (ERβ)	ESR2	Bindding DB
Estrogen-related receptor alpha	ESRRA	CHEMBL
Thrombin	F2	TCMSP、Bindding DB
Coagulation factor VII	F7	TCMSP

Table S2 Kaempferol target list (Continued)

Gene	Abbreviation	Database
Enoyl-acyl-carrier protein reductase	FASN	Bindding DB
Flap endonuclease 1	FEN1	CHEMBL
Tyrosine-protein kinase receptor FLT3	FLT3	Bindding DB、CHEMBL
Gamma-aminobutyric acid receptor subunit alpha-1	GABRA1	TCMSP
Gamma-aminobutyric-acid receptor alpha-2 subunit	GABRA2	TCMSP
AXL receptor tyrosine kinase	GAS6	Bindding DB
Beta-glucocerebrosidase	GBA	CHEMBL
Glyoxalase 1	GLO1	Bindding DB、CHEMBL
Glycogen Synthase Kinase-3, beta	GSK3B	Bindding DB
Glutathione S-transferase Mu 1	GSTM1	TCMSP
Glutathione S-transferase Mu 2	GSTM2	TCMSP
Glutathione S-transferase P	GSTP1	TCMSP
Hyaluronan synthase 2	HAS2	TCMSP
Hypoxia-inducible factor 1 alpha	HIF1A	CHEMBL
Heme oxygenase 1	HMOX1	TCMSP
Aldehyde dehydrogenase 1A1	HPGD	CHEMBL
Estradiol 17-beta-dehydrogenase 1 (17beta-HSD1)	HSD17B1	Bindding DB、CHEMBL
Endoplasmic reticulum-associated amyloid beta-peptide-binding protein	HSD17B10	CHEMBL
Estradiol 17-beta-dehydrogenase 2	HSD17B2	Bindding DB、CHEMBL
Heat shock protein HSP 90	HSP90AA1	TCMSP
Intercellular adhesion molecule 1	ICAM1	TCMSP
Isocitrate dehydrogenase [NADP] cytoplasmic	IDH1	CHEMBL
Insulin-like growth factor I receptor	IGF1R	Bindding DB
Inhibitor of nuclear factor kappa-B kinase subunit beta	IKBKB	TCMSP
Insulin receptor	INSR	TCMSP
Transcription factor AP-1	JUN	TCMSP
HERG	KCNH2	CHEMBL
Calcium-activated potassium channel subunit alpha-1	KCNMA1	CHEMBL
Lysine-specific demethylase 4A	KDM4A	CHEMBL
Lysine-specific demethylase 4D-like	KDM4E	CHEMBL
Prelamin-A/C	LMNA	CHEMBL
Interstitial collagenase	LSS	TCMSP

Table S2 Kaempferol target list (Continued)

Gene	Abbreviation	Database
Monoamine oxidase	MAOA	Bindding DB
Mitogen-activated protein kinase 8	MAPK8	TCMSP
Microtubule-associated protein tau	MAPT	CHEMBL
Hepatocyte growth factor receptor	MET	Bindding DB
alpha-Glucosidase (α-Glucosidase)	MGAM	Bindding DB
DNA-3-methyladenine glycosylase	MPG	CHEMBL
Myeloperoxidase	MPO	CHEMBL
Nuclear receptor coactivator 2	NCOA2	TCMSP
Sialidase	NEU1	Bindding DB
Sialidase 2	NEU2	CHEMBL
Nuclear factor erythroid 2-related factor 2	NFE2L2	CHEMBL
Nitric oxide synthase, inducible	NOS2	TCMSP
Nitric-oxide synthase, endothelial	NOS3	TCMSP
NADPH oxidase 4	NOX4	Bindding DB、CHEMBL
Bile acid receptor FXR	NR1H4	CHEMBL
Nuclear receptor subfamily 1 group I member 2	NR1I2	TCMSP、STITCH
Nuclear receptor subfamily 1 group I member 3	NR1I3	TCMSP
Glucocorticoid receptor	NR3C1	CHEMBL
Progesterone receptor	PGR	TCMSP
PI3-kinase subunit gamma	PIK3CG	TCMSP、Bindding DB
Serine/threonine-protein kinase PIM	PIM1	Bindding DB、CHEMBL
DNA polymerase eta	POLH	CHEMBL
DNA polymerase iota	POLI	CHEMBL
Serum paraoxonase/arylesterase 1	PON1	CHEMBL
Peroxisome proliferator-activated receptor alpha	PPARA	CHEMBL
Peroxisome proliferator-activated receptor delta	PPARD	CHEMBL
Peroxisome proliferator-activated receptor gamma	PPARG	TCMSP、CHEMBL
Serine/threonine-protein phosphatase 2B catalytic subunit alpha isoform	PPP3CA	TCMSP
Prolyl endopeptidase	PREP	CHEMBL
Trypsin-1	PRSS1	TCMSP
lens epithelium-derived growth factor p75	PSIP1	Bindding DB
26S proteasome non-ATPase regulatory subunit 3	PSMD3	TCMSP
Prostaglandin G/H synthase 1	PTGS1	TCMSP、CHEMBL
Receptor-type tyrosine-protein phosphatase S	PTPRS	CHEMBL

Table S2 Kaempferol target list (Continued)

Gene	Abbreviation	Database
Rac GTPase-activating protein 1	RACGAP1	CHEMBL
Transcription factor p65	RELA	TCMSP
Nuclear receptor ROR-gamma	RORC	CHEMBL
Ribosomal protein S6 kinase alpha-3	RPS6KA3	STITCH
Retinoid X receptor alpha	RXRA	CHEMBL
E-selectin	SELE	TCMSP
Sucrase-isomaltase	SI	Bindding DB
Glucose transporter	SLC2A1	CHEMBL
Solute carrier family 2, facilitated glucose transporter member 4	SLC2A4	TCMSP
Sodium-dependent noradrenaline transporter	SLC6A2	TCMSP
Antileukoproteinase	SLPI	TCMSP
Superoxide dismutase	SOD1	CHEMBL
SRC	SRC	Bindding DB
Signal transducer and activator of transcription 1-alpha/beta	STAT1	TCMSP
Synapsin-1	SYN1	Bindding DB
Tyrosyl-DNA phosphodiesterase 1	TDP1	CHEMBL
Tumor necrosis factor	TNF	TCMSP
DNA topoisomerase II alpha	TOP2A	CHEMBL
DNA topoisomerase II	TOP2B	TCMSP
Cellular tumor antigen p53	TP53	CHEMBL
TTR	TTR	Bindding DB
Tyrosinase	TYR	Bindding DB、CHEMBL
UDP-glucuronosyltransferase 1-3	UGT1A3	STITCH
UDP-glucuronosyltransferase 1-7	UGT1A7	STITCH
UDP-glucuronosyltransferase 1-8	UGT1A8	STITCH
UDP-glucuronosyltransferase 1-9	UGT1A9	STITCH
UDP-glucuronosyltransferase 3A1	UGT3A1	STITCH
Ubiquitin carboxyl-terminal hydrolase 1	USP1	CHEMBL
Vascular cell adhesion protein 1	VCAM1	TCMSP
Xanthine dehydrogenase	XDH	Bindding DB、CHEMBL
Xanthine dehydrogenase/oxidase	XDH	TCMSP
Inhibitor of apoptosis protein 3	XIAP	CHEMBL

Note: there are 159 kaempferol targets in total
